# Winter Health Resorts

**Published:** 1898-10-08

**Authors:** 


### WINTER HEALTH RESORTS.

Specially Compiled for the Autumn Special Number of "The Hospital."

The people whom we meet with at health resorts may be roughly divided into three main classes: (1) At the one extreme we have the mere holiday wanderers, who are in search of change and mental relaxation rather than of either bath or climatic treatment. (2) At the other we find those who visit health resorts gravely and seriously, for the sake of undergoing some "cure" or "course," be it of baths or waters, of "exercises," or of massage. Both these classes crowd the watering-places of England and the Continent—especially the latter—during the summer season, partly because summer, when the days are long and outdoor life is pleasant, is the natural time for holiday-making; and partly because hydro-therapeutics, the taking of baths, the drinking of waters, and the various eliminative processes which go to constitute Spa treatment, are carried out with best effect at a time of year when moderate exercise can be taken and fresh air can be enjoyed without risking any sudden check to the action of those emunctory organs, the stimulation of which is the real essence of so much of the bath and water treatment which is employed at "watering-places" properly so-called. (3) Between these extremes, however, are great numbers of people who frequent health resorts because the climate and mode of life met with at such places suit them better than their home surroundings, and among this class are to be found the majority of those who fill the winter health resorts.

Of those who are driven for health reasons to leave their homes in winter, the great majority are not so much in search of a cure as of some safe haven where they may lie protected from the rigours of the season until the summer comes round once more, and, even when some definite line of treatment is prescribed, treatment in winter must always be largely a matter of climate. In the choice of a winter health resort, then, climate is the main factor and is the thing to be first considered; yet it must never be forgotten that climate is not everything, and that just in proportion as people are more seriously ill, so is it increasingly necessary that they should not cut themselves adrift from the comforts to which they have been accustomed at home, nor suddenly change the habits and routine of their home life. To the strong and those who retain a fair amount of vigour, mere change is delightful and often beneficial, but to the invalid it is otherwise. He has gradually selected all his surroundings so as to suit himself; if these were changed, even while at home, he would be upset, and the best that can be done for him, in many cases, would be—if that were possible—that he and all his *entourage* should by a stroke be transplanted to his new place of abode, the change being confined to climate alone.

Among the factors which go to produce the climate of a locality are: Distance from the equator, altitude, distance from the coast, prevalence of wind and the direction of the prevailing winds, degree of saturation of the air with moisture, prevalence or absence of rain or snow or fog, situation in regard to the sun, clearness of the atmosphere, length of day in winter, diurnal range of temperature, and many other things, some of which affect large areas, while others are purely local. It is clear, then, that when so many influences are engaged, even places which are quite near to each other may vary greatly in their climate. This is frequently seen in Alpine valleys, where a slight change of position may bring one into a climate totally different. One side of the valley may be exposed to the sun throughout the whole length of the winter's day, while the other may never see the sun from the end of September till the beginning of April; one spot in a valley may be chilled every night by icy winds pouring down the mountains, while another jutting out on a spur of the hill may be practically in a calm the night through. It is, then, of little use theorising as to the sort of climate which is likely to exist at any given place. We must take the experience of those who have been there, and consider how far the sort of weather and the other climatic conditions which are known to be usual at given places at given times of the year are likely to suit the particular case we have in hand.

Taking the health resorts which are at the disposal of invalids during the winter, they may be divided as follows: Mountain, marine, and inland.

*Mountain Health Resorts.*—The mere elevation of a district is but one of many factors in the production of its climate, still the altitude often becomes the leading influence, especially in regard to the treatment of consumption. In Europe the treatment by high altitudes practically means a visit to one or other of a few places in Switzerland, where we may expect to find (*a*) a low barometric pressure, which is one at least of the causes of that deeper respiration and greater expansion of the lung which are by some looked upon as essential parts of the altitude cure of phthisis; (*b*) great transparency of atmosphere, not only to light, but to the heat and actinic rays of the sun; (*c*) low temperature, except actually in the sunshine, as a consequence of which, and of the fact that during the winter the whole district is deeply covered with snow, the air is practically sterile; (*d*) absence of fog; (*e*) a large amount of sunshine compared with the length of the day; (*f*) dryness of the air; (*g*) and, in the places selected as health resorts, rareness of wind. On first arriving at such a place various disturbances of function are experienced, but after a time acclimatisation takes place, and then it will be found that the main alterations which persist, as compared with the previous condition, are deeper and fuller respirations, with an increased exhalation of carbonic acid and of watery vapour, a distinct enlargement of the thorax, improved appetite and assimilative power, a greater sense of vigour, an increase in the number of red blood corpuscles, and probably an increased power of resistance to the action of micro-organisms. The effects of residence at a considerable elevation may then be considered as in part vital and in part physical. So far as the vital changes are concerned, they seem to be due to the large amount of time which is passed in the open air at these health resorts, to the tonic action of the cold, dry air,

### WINTER HEALTH RESORTS—(*continued*).

to the purity of the atmosphere, and to the large amount of sunlight; and, so far as these are concerned, they do not differ materially from the effects which are obtainable in many places at lower elevations. In addition to these effects, however, there are also certain specific changes in the lungs and the thorax which are peculiar to high altitudes, and are, in the opinion of some, of very great importance in the curative action of these places so far as phthisis is concerned, and also in rendering permanent such amelioration as may take place. Elevated districts in other parts of the world produce many of the same effects with this exception, that in those places which are situated nearer to the equator an equal elevation does not produce the same coldness of the air, nor does it lead to the district being covered with snow, so that it sometimes happens that the air, although equally rarefied, is by no means as sterile as is the case in the snow-covered Alps. Moreover, in some places there is apt to be a good deal of dust whenever there is any wind. It must also be remembered that in some places, as in the hill stations in India, the periodical winds are at times highly charged with moisture, which is in many cases injurious. In all cases, however, the same enlargement of the chest and the same thorough opening up of the air cells takes place as are such marked consequences of residence in the high Alps.

*Marine Resorts.*—These include sea voyages. The climate of the ocean is marked by the comparative moisture of the air which is continuously present, the equability of its temperature, and its freedom from all germs; seaside places partake of these peculiarities, but sea voyages are characterised, in addition, by the opportunities they give for rest and mental repose—torpor, indeed—so that while they are often found to be distinctly stimulating to the appetite, even sometimes to the length of causing indigestion, they are sedative to the nervous system. The great disadvantages attending them is the chance of bad weather and the difficulty in getting exercise. The effects of seaside climates are to improve the appetite and the assimilative powers, and in many cases to produce sleep. There is, however, a very great difference in the effects of different seaside resorts, and it is quite evident that many other circumstances besides their mere position on the coast have to do with their varying action on different individuals. Among these probably the amount of moisture in the air is one of the most important, and it is usual to divide seaside resorts into the humid and relaxing and the dry and bracing—a division, however, which is somewhat arbitrary, and does not always hold good throughout the year.

*Inland Health Resorts.*—Of winter health resorts comparatively few are situated inland, except those which, as in Egypt, in the Karoo in South Africa, and in some parts of Australia, depend for their efficacy upon the dryness and purity of their atmosphere.

### RESORTS FOR SPECIAL DISEASES.

In deciding upon a winter resort for an individual case, one has to put to oneself this question, What is the object of this patient in leaving home? Is the visit to a health resort to be made for the sake of treatment, for the specific effect of a particular climate, of its altitude, its dryness, or what not; or is it merely for the sake of escaping from the evil effects of an English winter? In the former category one places cases of convalescence from illness; many cases of anæmia in town dwellers; most cases of nervous breakdown; cases of "scrofula" in children, and of bronchitis with excessive secretion in adults; cases of tendency to tuberculosis and all cases of curable phthisis. These cases require change or climatic or sanatorium treatment quite irrespective of the season, and the choice of resort depends upon the disease and upon the effect which it is desired to produce. In other cases, however—and these probably the majority of the frequenters of winter resorts—the patient gets on pretty well in the summer, but fails in the winter, and what one wants is merely a protective climate, a health resort in which one can escape the evils of the winter rather than one which gives any specific benefit. These cases are very numerous, and include especially chronic bronchitis, emphysema, Bright's disease, gout, chronic rheumatism, people who are old or feeble, those who cannot bear the cold, and a large number of cases of phthisis, some of which, although they have recovered as far as may be, find that they "cannot stand the winter," while others who, although so far gone that they cannot hope for cure, yet are able to live on by so arranging their place of residence that they shall pass their time in perennial summer. There is nothing specific about the treatment of such patients; a large number of resorts are open to them, and what they have to do is principally to find a place which shall enable them to escape the rigours of the winter. To a large extent then moderate warmth is the main characteristic of what may be termed the protective health resorts. But even here a careful choice has to be made, for in some places warmth is associated with dryness and in others with an over-abundant moisture, in some there is great equability while in others of perhaps the same mean temperature certain hours are apt to be very chilly, as occurs along the Riviera about sunset, and in Egypt during the night.

Without attempting any minute classification, but always bearing in mind the distinctions we have drawn as to the aims and objects of climatic treatment, it will be well to indicate in a general manner the sort of climates which are found useful in the main groups of diseases in which visits to winter health resorts are commonly recommended.

#### Anæmia, including Chlorosis.

When anæmia does not mean chlorosis the treatment of the condition itself is to a large extent merged in that of the disease by which the deficient sanguinification is produced, such as phthisis, dyspepsia, malaria, &c. But anæmia, from whatever cause it may arise, leaves the patient less able than usual to resist adverse influences, and thus it is often very desirable to pass the winter in a protective climate. Warm, sunny seaside resorts are, then, very useful in many cases of ordinary anæmia, as they are also in those cases of chlorosis in which debility is marked, and much rest is required. Often, however, these cases require a more tonic influence, such as is to be obtained at places of moderate elevation, and (where there is sufficient

### WINTER HEALTH RESORTS—(*continued*).

strength to enable patients, even for a short time each day, to participate in the outdoor amusements which are in vogue at mountain resorts) the high altitudes suitable for phthisis are useful in many slight or recovering cases, and especially in such cases of anæmia as result from malaria. Where, however, anæmia occurs as a symptom, the main disease should have the first consideration. In regard to chlorosis, it must be remembered that medical treatment is generally necessary, and that it is of the extremest importance that too much exercise should not be taken until the cure is well advanced. Among the few ferruginous waters available during the winter may be mentioned Harrogate and Bagnères de Bigorre; but we may be allowed to doubt whether the existence of an iron water at any spa should be taken into serious consideration in the choice of a winter resort. Facilities for obtaining sunshine and fresh air are of far more importance.

### Bright's Disease.

In the winter months, patients suffering from Bright's disease are safest in warm, sheltered seaside places in which the air is dry, such as the Riviera, or in Egypt. Much, however, depends on the stage of the malady. Some cases do well in the moister climate of Madeira, or, as an intermediate, in Algiers. If at all advanced, the fatigue of a long journey is a serious consideration, and it may be better to go to Bournemouth, Torquay, Sidmouth, Falmouth, or other places near home than to go abroad. Although moderate altitudes are often useful in summer, the high Alpine resorts are not suitable for cases of albuminuria.

### Chronic Catarrh.

Chronic catarrh of the air-passages (nasal, naso-pharyngeal, bronchial, or pulmonary) often demands that the patient shall escape from the cold and damp of an English winter, although great relief is often experienced at Bournemouth, Clifton, Southport, Grange, and other sheltered places on the English coast. The drier seaside resorts, such as the Riviera, and desert climates, such as that of Upper Egypt, are generally the most useful; but in some cases in which the expectoration is scanty and cough is troublesome, the more humid climates, such as Madeira and the town of Algiers or Torquay, or the south-west of Ireland, are to be preferred. If proper care is exercised the Riviera is peculiarly suitable for many cases of chronic laryngitis, and is, speaking generally, the best place to which to send cases of laryngeal phthisis, although early cases may sometimes go to Egypt with advantage. Laryngeal cases do not as a rule do well in the high altitudes. Mere hoarseness in a case of phthisis need not be looked upon as a bar to sending such a case to the mountains, but if the arytenoids are involved, or if there are signs of the presence of perichondritis, they should not be sent there. Cases of laryngeal ulceration should certainly not be sent to Egypt, but often derive comfort from the climate of Mentone or San Remo.

### Diabetes.

Various waters are recommended in the treatment of this disease; they are, however, chiefly taken as summer "courses." During the winter protective climates are to be selected, mild, sunny localities, but not so hot as to prevent a fair amount of exercise being taken. Mental change and occupation is also very desirable. Hence Egypt, Algiers, Rome, and the Riviera are useful if care be taken to avoid chill. Yachting in the Mediterranean is an especially useful agency, affording as it does a great amount of change, while not of necessity interfering with diet or cutting the patient adrift from his culinary arrangements. The glycosuria of fat people may often be usefully considered as a side issue of the gouty diathesis.

### Dyspepsia.

This is often the result of overwork and nervous strain, and is much relieved by lazy travel. But many dyspeptics are very susceptible to cold, and in such cases travel should be confined to moderately warm climates. Where, however, dyspepsia is connected with organic disease, or has produced much asthenia, passing the winter in a well-protected locality is likely to do the best. To that large class of persons who, without being seriously ill, cannot stand the cold, and especially to many elderly persons with gouty tendencies who cannot, during an English winter, get the exercise, by the aid of which when they were younger they used to keep the gout at bay, the question of spending the winter travelling about in India deserves serious consideration. The climate in winter is delightful; no place can offer greater change of scene, while the social attractions are considerable, and English requirements are thoroughly catered for in all the larger places, to which alone such a tourist is likely to proceed.

### Heart Disease.

When heart disease is well compensated, it does not seriously complicate conditions with which it may co-exist, except that it is well not to send such patients to high altitudes. When, however, failure is taking place, a quiet, protective, well-sheltered resort is best in winter. Bournemouth, Southport, Worthing, and the Riviera are often useful. But it must be remembered that failure is often due to the presence of some intercurrent condition, such as Bright's disease, or bronchitis, or emphysema, or pulmonary congestion, and that the climate which is most suitable for the complication, whatever it may be, will often give the greatest relief to the failing heart. In this way even the longer journey to Egypt, if taken with all precaution, may give great relief by removing bronchitic complications.

### Phthisis.

**Alpine Resorts.**—The best cases for the mountains are the early ones, whether unilateral or bilateral, so only that the disease is limited, and the pyrexia moderate, and the physical power such that an outdoor life can be borne. Pyrexia, however, that is obviously due to septic absorption, is not of such importance in this respect as that which arises from the tuberculous process. Dr. Theodore Williams says: "tubercular consolidation does best, the large proportion of cases resulting in complete recovery." Patients over 40 do not benefit so much as those under that age.

Hæmorrhage in the early stage of phthisis is no bar to mountain treatment, but the hæmorrhage which is associated with larger excavations, a form which is no doubt usually produced by aneurismal conditions of the

**WINTER HEALTH RESORTS**—*(continued)*.

pulmonary vessels, should certainly be considered to contra-indicate treatment at high altitudes.

Patients, again, whose breathing surface is much reduced by disease, so that they readily show symptoms of lividity, dyspnœa, and inability to keep warm, are not fit subjects. We must also exclude cases of double cavities and all advanced forms of pulmonary tuberculosis, and also cases of acute phthisis.

Cases in which there is albuminuria certainly should not be sent, and the same applies to all cases in which there is valvular disease of the heart or degeneration of the arteries.

Diarrhœa, if apparently caused by tuberculous ulceration, should be held to negative mountain treatment, as also should laryngitis, if the arytinoids are affected, but not necessarily if merely catarrhal.

There is one sort of case which certainly should be sent, if possible, viz, cases in which phthisis threatens after pleuritic effusion. Such cases are often markedly benefited.

Places in repute for the treatment of phthisis by high altitudes in the winter are:—In Switzerland: Davos (5,200 ft.), St. Moritz (6,000 ft.), Leysin (4,712 ft.), Arosa (6,100 ft.), Les Avants (3,500 ft.).

In the Rocky Mountains: Denver (5,000 ft.), a large town of 150,000 inhabitants; Colorado Springs (6,022 ft.), a town of 15,000 inhabitants, but almost exclusively a health resort; Glenwood (5,000 ft.), on the western slope, and not so dry as on the other side.

Denver and other places in Colorado in the United States, are especially advantageous in early cases, and as a prophylactic measure, after pleurisy, for example. They are especially useful where prolonged residence at high altitudes is necessary, because there are opportunities for settling and earning a living.

In the Andes: Huancayo (10,000 ft.), and Jauja (10,000 ft.), both in Peru; Quito (9,500 ft.), in Ecuador, a large town of 80,000 inhabitants; Santa Fé de Bogata (8,648 ft.) in New Granada. (See also page 32.)

**Desert Climates.**—(See page 29).

**Australia.**—Early cases sometimes do well in Australia, but whether they do as well as they would at high altitudes is a question. Quiescent cavity cases, again, receive benefit, both by the voyage and also afterwards. It is essential, however, to go up the country, and not to linger in the coast towns.

**South Africa.**—The same is true of South Africa; but in many of the parts where most benefit can be obtained the life is rough and comfort, even if obtainable, is very expensive. None should go unless in a fairly vigorous condition as to general health, and possessed of some resources on which to draw in case of failure. Cases with cavity hæmoptysis should certainly not go. It should be borne in mind that the parts of South Africa which are most suitable for phthisical cases are the great elevated plains, or interior plateaux, situated behind the mountains, and that these plains themselves stand so high that they may almost be regarded as mountain resorts. Their great characteristics are intense radiation and great dryness. But the nights are cold, and warm clothing is necessary. To some nervous people who are susceptible to "electrical" conditions of the atmosphere, the terrific thunderstorms which so frequently occur detract greatly from the pleasure of residence on these high plateaux.

There is no doubt that the climatic conditions which are available in South Africa are likely to be useful for the treatment of many forms of debility, the result of the strain of civilised life. Still, it is for cases of tuberculosis that South Africa is in the highest repute, and for such the following places are of most importance: Craddock (2,855 ft.), Beaufort West (2,792 ft.), Burgersdorp (4,552 ft.), Tarkastad (4,280 ft.), Aliwal North (4,348 ft.), Bloemfontein (4,500 ft.), and Harrismith (5,250 ft.) Kimberley (4,300 ft.) is one of the most populous and important towns of the Cape Colony. It contains a general hospital, and the sanatorium which has been erected by Mr. Rhodes is a well-appointed place, and is likely to prove useful to many an invalid.

**Sea Voyages.**—In the treatment of phthisis sea voyages suit, especially, quiescent cavity cases. (See page 29.)

**Seaside Resorts.**—*The Riviera* (See page 28).—Cases complicated with catarrh and bronchitis, or with attacks of catarrhal pneumonia, and cases of laryngeal phthisis are much relieved, or, at any rate, made more comfortable by wintering on the Riviera, but early cases also often do well if a proper régime is followed. Even advanced cases of phthisis, in which cure is out of the question, may often enjoy the remainder of their lives much better, if they can afford all the comforts they require, by spending the winter on these southern shores.

*The English South Coast.*—(See page 27).

*Algiers.*—(See page 29.)

*Madeira and the Canaries* are useful in cases of febrile irritability and dry cough, but they are both somewhat relaxing. The climate of Funchal is remarkably equable, and there is a complete absence of that sudden chilliness about sunset which is so dangerous a feature at many places. To many people the climate is enervating and relaxing, but it is useful in bronchial and laryngeal catarrh, and in cases of emphysema and albuminuria. Unfortunately, it is very hilly, which makes it difficult to get about on foot. Teneriffe is decidedly drier than Madeira. Orotava is the principal place for invalids.

**The Sanatorium Treatment of Phthisis.**—Of late years a great movement has taken place having for its object the institution of sanatoria for the treatment of consumption, provided with all the appliances and arrangements which are found necessary for maintaining the purity of the air. It has become pretty clear that the one thing which is common to all the various methods which have had their vogue in the treatment of consumption has been the purity of the air breathed, and that the success of the different methods has depended largely upon the length of time the patients have passed absolutely in the open. Hence has arisen the "open-air" treatment of consumption, for the carrying out of which arrangements have been made for patients to live during the day in sheds and verandahs, and even when they come indoors at night for their bedrooms to have widely-open windows, and it has been found that this mode of treatment can be carried out during the winter even in England. Moreover, it has been made evident that this mode of

### WINTER HEALTH RESORTS—(continued).

treating phthisis is applicable to the more acute cases, and is almost a specific in dealing with the pyrexia which often is so marked a feature of the more or less acute onset of the disease. Thus the open-air treatment is likely to take a very important place in the treatment of many cases which are at present cut off from all chance of removal to the health resorts where the mountain cure is carried on. To those who live in good surroundings, with fair-sized gardens, on a dry soil, who have money and servants, and can afford time and give thoughtful supervision, the open-air treatment may perhaps be managed at home; but to the great mass of people (especially until the essentials of the method have been a little better worked out than is the case at present) the open-air cure will be best carried on at institutions, of which many are now coming into existence. In regard to this point, it is especially to be borne in mind that the sanatorium treatment involves something very much more than merely putting a patient in the open air, and leaving him to take his chance. In the best conducted sanatoria the most careful supervision is exercised over every detail of the patient's life, and both exercise and diet, as well as the amount of open air, are exactly regulated according to the varying condition from day to day. One must in this matter recognise the claims of the specialist, and if we are to do the best for our patients, we must be prepared to admit, what is undoubtedly true, that residence in a sanatorium, such for example as the one at Nordrach, where a small number (40 to 50) of patients are under the constant supervision of a specialist who is entirely devoted to their care, is likely to give better results than residence in the most perfectly appointed lodgings or hotel in the finest possible climate, under the direction of a physician, whose visits are only occasional, or, as the patient would say, "when they are required." At present the treatment is mostly carried on at Nordrach, Falkenstein, Goerbersdorf, Hohenhonnef, Ruppertshain, Reiboldsgruen, St. Blasien, in Germany; Davos, Leysin, and Arosa, in Switzerland; Canigou, in France; Bournemouth, Cromer, and the Ventnor Consumption Hospital, in England; Quarrier's Consumptive Hospital for Women, Renfrewshire; and the Craiglieth Consumptive Sanatorium, near Edinburgh, in Scotland.

There is no doubt, however, that in a very short time, as patients come forward willing to undergo the restrictions involved in the sanatorium treatment, sanatoria will be established in many places. (See also page 32.)

### Rheumatism, Gout, Sciatica, &c.

So far as the chronic forms of these affections are concerned their winter climatic treatment runs on much the same lines. Change of scene, dry and sunny climates, where as much exercise as the local complications will allow can be taken, are generally the best.

Many baths are useful for rheumatism and for chronic gouty conditions, but comparatively few are used during the winter. Of the latter, however, may be mentioned Bath, Sidmouth, Droitwich, Matlock, Strathpeffer, Buxton, Harrogate, Wiesbaden, Aix-le-Bains, Vernet-les-Bains, Kreuznach; but at none of these places is the bath treatment either so satisfactory or so safe, in the majority of cases, during the winter as in the summer season. Among protective winter stations may be mentioned Bournemouth, Torquay, Clifton, Ajaccio, Algiers, Mentone, Monaco, and other sheltered spots on the Riviera, and, where dryness is sought, Egypt. Teneriffe and Madeira also suit some cases very well.

Gouty people, when not actually suffering, are often impatient of the regimen of health resorts, and comfortably-arranged yachting, with visits to interesting places in warm latitudes, is a good way of getting through the cold months. Long voyages are, however, apt to disagree in consequence of the difficulty of getting exercise.

### Rickets.

The use of climate in rickets is principally to ensure that the patients shall get plenty of fresh air, which in cold and damp places they cannot obtain. Warm, sheltered seaside places are the most suitable in winter, among which the Channel Islands may be especially mentioned.

### Syphilis.

There is nothing to prevent the combined bath and specific treatment which is given at Aix-la-Chapelle, Wiesbaden, or Luchon being carried out in the winter; but any protective, mild, dry, and sunny climate, combined with such medicinal treatment as may be necessary, may be resorted to during the winter months. Syphilitics often cannot bear cold or much fatigue.

---

### ENGLISH HEALTH RESORTS.

We cannot find in England what we can on the coasts of the Mediterranean or in the Canary Islands, neither the brightness, the warmth, nor the long-continued fine weather. But in places on the English coasts we can find a climate so vastly superior to what many patients would have to put up with at home; we can get to these resorts so easily, and, if they do not suit, we can get back again with such facility; we are all the time so near our friends; and we may, if we choose, be all the time so surrounded with the comforts to which we have become accustomed that only naturally the English south coast deserves the first consideration of those who are in search of a winter health resort.

The characteristics of most of the places we shall have to mention are that they have mild winters and cool summers; that they have a rather humid air with many rainy days; and, unfortunately, that on these days the rain is apt to be spread out through many hours. The solar radiation is less intense than on the Mediterranean coasts, but so also is the radiation at night, so that the chill at sunset is less than, for example, on the Riviera. Dividing the coast of England roughly we may speak of the west as warm and moist and the east as dry and cold. In times past the choice of winter resorts had such exclusive reference to chest complaints and especially to consumption, and cold was then looked upon as so injurious that the east coast of England was quite neglected. But the east coast, although cold, is often bright and sunny in the winter, and many cases of debility and of that tendency towards local tuberculosis which goes by the name of scrofula gain much advantage from such places as Margate, Hunstanton, Cromer, and many others which, however icy they may be, are bright and clear. Coming to the south coast, Hastings and St. Leonards, together with

### WINTER HEALTH RESORTS—*(continued).*

Eastbourne and Brighton, are somewhat intermediate, both geographically and climatically between the east and the south-west resorts, and are very useful for many atonic cases. Brighton in the autumn up to Christmas; Hastings until the east winds come. The Undercliffe on the Isle of Wight, Boscombe, and Bournemouth are all in parts well-protected winter climates, and do admirably for many cases of bronchitis, for elderly people and young children, and also for such cases of incipient phthisis as cannot go further.

Then we come to the warm and equable climates of Sidmouth, Salcombe, Budleigh-Salterton, Dawlish, Teignmouth, and especially Torquay. For purely protective climates this is probably the most favoured part of England, and although the east wind is nowhere entirely avoided, many feeble cases of bronchitis, emphysema, kidney disease, and dyspepsia, which in winter suffer from lack of fresh air and exercise when at home, do very well in these more sheltered spots. Falmouth must also be included in among these protective resorts. Pwllheli, Llandudno, Grange, and Southport are useful marine climates; while on the west coast of Scotland and the south-west of Ireland there are several places which, from the action of the Gulf Stream, have remarkably mild although moist winters, such as Rothsay, Glengariff, and Queenstown.

The Channel Islands are warm and bright, but are somewhat moist and much exposed to wind. They are especially useful for strumous and ricketty children. Elderly and feeble people do well until the east winds come in March.

More inland in England there is Clifton, which is especially resorted to in the autumn and the winter by cases of hepatic disorder, bronchial, pharyngeal, and laryngeal catarrh, and chlorosis. The educational facilities render it very useful in many cases of delicacy in childhood. Bath, Malvern, Droitwich, Buxton, Harrogate, and Strathpeffer are to some extent resorted to during the winter for special treatment chiefly in connection with chronic forms of gout and rheumatism; and those of them which stand fairly high, such as Malvern, Harrogate, and Buxton, for their pure, clear air, which is useful in some cases of debility and anæmia. Unfortunately, at all these places spells of bad weather are not uncommon.

### THE MEDITERRANEAN.

Various as are the climates met with on the Mediterranean coasts, they all to a greater or less extent are under the influence of what is the great peculiarity of that sea, namely, its warmth, for down to its deepest depths the water of the Mediterranean is of a temperature of from 54 deg. to 56 deg. Fahr. The injurious influences on these coasts are mostly connected with the winds which tell with especial force on those winter health resorts which lie along the Riviera.

#### The Riviera.

This part would, indeed, be an almost perfect haven of peace but for the winds. The mistral or north-west wind blowing across the cold plateau of Central France, is very disagreeable. It occurs chiefly in March, but comes occasionally throughout the winter. The sirocco is hot and enervating, but comes only occasionally during the winter. The greco or north-east wind is described as "a biting, cold wind, often accompanied with sleet, hail, or snow." Fortunately, in the Western Riviera it is not so much felt as it is further eastwards. The choice, then, of a winter resort on the Riviera to a large extent hinges on the prevalence of one or other of these winds, some places being very much better protected than others. Speaking generally, one may say that the climate of the Riviera is characterised by dryness, abundant sunshine, and complete absence of fog, but that its effects upon the nervous system are of an "exciting" nature, so that it may be harmful to hysterical and highly-strung individuals, who, under its influence, are apt to lose their sleep. For those who wish to escape an English winter the Riviera presents every facility, the gaiety of the life in some of the towns being a source of much attraction. The real invalid, however, ought to submit to a careful regimen, the reasons for which do not always at first sight seem very apparent, and much mischief results in some cases from the temptations which fall in the way of sick folk.

The following health resorts along the Riviera may be mentioned, but there are many other places where accommodation may be obtained:—

*Hyères.*—Situated three miles from the sea; somewhat windy and dusty, but not quite so "exciting" to neurotic patients as some other places which are closer to the sea.

*Cannes.*—Now a large district. A good bracing climate.

*Grasse* stands high, and is cooler than Cannes.

*Antibes.*—Not very well sheltered.

*Nice.*—Hardly a health resort, but a very good place for those who require change. Often dusty and windy.

*Cap Martin.*—Very good; free from dust. More sedative than Mentone or Monte Carlo, but as yet a pleasure rather than a health resort.

*Monte Carlo.*—Delightful place, and one which, from a mere health point of view, possesses very many advantages. Their compulsory idleness, however, renders invalids a ready prey to the attractions of the gaming tables.

*Mentone.*—In a manner the type of the climate of the Riviera. Very well protected in the eastern portion.

*Bordighera.*—More open and bracing than Mentone; and the same may be said of San Remo and Alassio.

#### Sicily.

A most beautiful country. Moister than the Riviera, and not so well supplied with comforts. It is suitable for many cases of overwork, rheumatism, and general debility, as well as for nervous and excitable people, who are too much stimulated by the Riviera. There is now good accommodation at Palermo, Taormina, Acireale, Catania, and Syracuse. Palermo is rather moist, and does not suit some bilious people. Taormina (of which we give an illustration) is a most beautiful place, perched on a hill high above the sea.

#### Corsica.

Ajaccio is a beautifully situated town in a fine bay facing south-west and protected from north and east by high mountains. It is a bright and sunny place, but is much moister than the Riviera, although not so damp

### WINTER HEALTH RESORTS—(*continued*).

as Madeira. There is fine scenery and very little dust. Good accommodation can be obtained, and the place would seem very suitable for cases of slow convalescence, for some cases of chronic rheumatism and gout which require a mild and sunny winter climate and yet do not bear much stimulation, and also for some asthmatics.

### Algeria.

*Algiers* is moister than the Riviera, drier than Madeira. It is subject to heavy falls of rain. The temperature does not fall so suddenly at sunset as on the Riviera, but as at Madeira the air becomes charged with moisture.

*Hamman R'Irha*, with its hot springs, stands 2,000 ft. above the sea, and may be resorted to when Algiers is too hot.

*Biskra* is practically a desert climate.

### DESERT CLIMATES.

Egypt and the Nile voyage may be taken as typical of desert climates; in fact, for European invalids there is practically no desert open besides Egypt, except Biskra on the edge of the Sahara in Algeria. The chief physical characteristics of a desert climate are "warmth, sunshine, dryness, freedom from rain, presence of ozone, and absence of germs of all kinds." Of these dryness is the chief peculiarity. All else can be got in other places, but dryness can nowhere be obtained in the same degree as in the desert. Occasionally there is dust, and dust storms undoubtedly are a trial, but they are not frequently met with by the health traveller if dusty regions are avoided. It must be remembered that the desert is not all sand or even pebbles, a great deal of it is rock. Hot as is the sunbaked desert, the winds are cold, especially in January, and the fall of temperature after sunset is very great. The cases of phthisis which are most likely to derive benefit from Egypt are: "(*a*) Cases complicated by bronchitis in which emphysema is also present; (*b*) cases of bronchiectatic phthisis, for which a winter or two in the Alps have already been prescribed, will often derive great benefit from Egypt; (*c*) cases for which the Riviera is too cold, or in which chills often recur; (*d*) cases of early consolidation in which, either owing to susceptibility to cold or some other reason, the Alps are contra-indicated; (*e*) cases with albuminuria in which the destruction of the lungs is not very great; (*f*) cases in which insomnia and nervous irritability form prominent symptoms." (Allbutt's System.) On the other hand, cases with intestinal ulceration or any tendency to diarrhœa, with laryngeal ulceration, and those beginning with acute pneumonic symptoms, should not be sent.

The physiological effects of the climate of Egypt may be described as bracing to the organism as a whole and sedative to the nervous system, so that it is useful in a large range of cases besides those of phthisis. It is of great value in gout and rheumatism, and in the early stages of senile cardio-vascular changes. All sorts of catarrhal conditions, whether of the bronchial, pharyngeal, or laryngeal mucous membranes, do well, as also do some cases of asthma, especially those which are kept up by the persistence of bronchial catarrh. Egypt is also very useful in cases of nervous exhaustion from over-work or worry, and in many cases of hypochondriasis and hysteria.

It must be borne in mind that Egypt is a very expensive place. To get the full benefit of a visit to Egypt one should go beyond Cairo, especially if the visit is made for the sake of the specific effect which the desert air exercises in disease of the lung. For those who seek merely for change of scene and to escape the winter, Cairo is a most interesting place, and a fairly long stay may usefully be made there; but real invalids and all tuberculous cases should keep out of the city as much as possible, and get up the river and into the desert.

The following are the places which are chiefly used as health resorts:—

*Mena House*.—From the middle of February onwards, as before that date there is too much moisture in the air, owing to the drying up of the plain of the delta. Good hotel.

*Helouan*, standing in an oasis in the desert about 10 miles from Cairo. As Helouan stands 150 ft. above the Nile Valley it may be utilised earlier, namely, from November. It is a very good place for early phthisis, and having desert air all around, and the desert itself being easily accessible. Good hotels.

*Luxor*, 450 miles south of Cairo, is a great centre for sight-seeing. There are good hotels, and comforts are accessible.

*Assouan* is somewhat more bracing than Luxor. It is drier and warmer but rather more windy, and, as a consequence, more dusty.

For those who can afford it camping out, keeping within reach of the river and thus of civilisation, ought to be one of the best things for phthisical cases, while the lazy life of the dahabeyah is perhaps the most soothing to those seeking relief from overwork.

The best time for arriving in Egypt is November, and most invalids should leave Cairo by the middle or end of April. They should not come straight home, but should rest awhile either in Italy or some warmish place.

### SEA VOYAGES.

The sea voyages which are usually undertaken by those threatened with consumption are (*a*) the steamship voyage to the Cape of Good Hope, which is really too short to be of much service; (*b*) the sailing voyage to Australia, which used to be very popular, but is now much out of fashion owing to the increased attractiveness of the steamboat service; (*c*) the steamship voyage to Australia, and (*d*) the steamship voyage to New Zealand. It is important to avoid returning by Cape Horn, and also to avoid the hot months in the Red Sea and the monsoon in the Indian Ocean. The advantages which steamers offer in regard to diet and fresh meat are far greater than can be found on sailing vessels.

Many others than consumptives, however, now find sea voyages very advantageous. Especially is this the case with the overworked, and with those requiring warmth and rest and freedom from responsibility—for even the responsibility of travel is a worry to some. These people sometimes find much benefit from a trip to the West Indies, and especially from the cruises in the well-equipped pleasure steamers which have of late years come so greatly into favour. Those which visit many ports in the Mediterranean are very useful in such cases.

The writer of this article wishes to acknowledge the assistance he has received from the following works: "The Mineral Waters and Health Resorts of Europe," by Hermann Weber and F. Parkes Weber; "Climate and Health Resorts," by Burney Yeo; various articles in "Allbutt's System of Medicine"; and the Special Tuberculosis Number of the *Practitioner*.

